# Personal Factors and Nutrition Intentions of Participants in a Nutrition Education Program for Limited-Resource Adults in Substance Use Recovery

**DOI:** 10.3390/nu18081304

**Published:** 2026-04-21

**Authors:** Omolola A. Adedokun, Brooke Jenkins, Jacqueline Corum, Jean Noble, Olumuyiwa Moses Desmennu

**Affiliations:** 1 Department of Community and Leadership Development, University of Kentucky, Lexington, KY 40506, USA; jacqueline.corum@uky.edu; 2 Family and Consumer Sciences Extension, University of Kentucky, Lexington, KY 40506, USA; brooke.jenkins@uky.edu (B.J.); jean.noble@uky.edu (J.N.); 3 Department of Pharmacy Practice and Science, University of Kentucky, Lexington, KY 40506, USA; moses.desmennu@uky.edu

**Keywords:** substance use recovery, nutrition intentions, self-efficacy, social cognitive theory

## Abstract

**Background/Objectives**: This exploratory, cross-sectional study examined the relationships between personal factors and the nutrition intentions of participants in Healthy Choices for Your Recovering Body (HCYRB), a nutrition education program for limited-resource adults in substance use recovery (SUR). **Methods**: The study used a single-sample survey design where HCYRB participants (*n* = 2163) completed a post-participation survey. Linear regression models were tested to assess the effects of personal factors such as nutrition knowledge, cooking skills, self-efficacy beliefs and current nutrition and physical activity practices on participants’ nutrition intentions. Variables were measured with a self-reported survey that participants completed after participation in HCRYB. **Results**: The final model (R^2^ = 0.39) showed statistically significant effects of self-efficacy beliefs, specifically, food resource management confidence and confidence to choose nutritious foods; current levels of water, soda, and energy drink consumption; physical activity level; and gender on nutrition intentions. **Conclusions**: Overall, the findings suggest that nutrition-related self-efficacy and current practices influence nutrition intentions of HCYRB participants. Future programs may focus on building participants’ nutrition-related confidence during SUR to enhance intentions and eventual behavior change. Such strategies may include programming activities that promote and affirm participants’ current positive nutrition-related behaviors (e.g., adequate consumption of water and involvement in physical activity). As participants master these healthy practices throughout the nutrition education experience, they will be more likely to gain confidence and motivation toward continuing the behavior throughout their recovery journey.

## 1. Introduction

Research has documented strong evidence of positive links between nutrition intentions and nutrition-related behavior [1,2,3,4]. Indeed, the first and most critical step in changing nutritional behavior is to form an intention to change one’s nutritional practices [5]. Given the critical role that intentions play in nutrition behavior, research studies have indicated associations between nutrition intentions and personal factors such as nutrition knowledge [6], self-efficacy beliefs, and self-confidence [5,7,8,9].

While research abounds on factors related to the nutrition intentions of adults in general, little is known about the nutrition intentions of adults in substance use recovery and the associated personal factors. This study addresses the gap in research by examining the relationships between personal factors and the nutrition intentions of participants in Healthy Choices for Your Recovering Body, a nutrition education program for limited-resource adults in substance use recovery (SUR).

The rise in substance use disorders in America continues to accelerate [10]. Individuals with chronic substance use disorders have a greater tendency to experience food insecurity, nutrient deficiencies, food cravings, malnutrition, poor diet quality, and increased body weight [11,12]. Additionally, adults in SUR often experience changes in food preference and consumption, including increased preferences for sweet and savory nutrient-poor foods [12].

The importance of nutrition education and dietary interventions in SUR cannot be overemphasized [11,13,14,15,16]. While research on outcomes of recovery-focused nutrition programs is limited [11], studies focusing on limited-resource populations in recovery are even scarcer [17]. The few available research studies indicate that nutrition education programs for adults in SUR are effective for improving fruit and vegetable consumption [18], fiber intake [19], and reducing consumption of calories from sweets and desserts [18] and sugar-sweetened beverages [20]. However, there is a paucity of research on the factors associated with the nutrition intentions of adults in SUR. The increasing focus on promoting sustained nutrition behavior during and after recovery brings with it a need to better understand the factors that facilitate the intention to change nutrition behavior among adults in SUR. In a study of food-related behaviors among low-income women in SUR, analysis of participants’ interview responses revealed that readiness to change is foundational to long-term nutrition behavior change [21]. Understanding the factors that facilitate or inhibit nutrition intentions of participants in their recovery journey has the potential to yield evidence-based findings to inform best practices for promoting lasting nutrition behavior in this audience.

The purpose of this study is to examine the personal factors associated with the nutrition-related intentions of participants in a nutrition education program for limited-resource adults in SUR. Specifically, the study examines the effects of nutrition knowledge and skills, self-efficacy beliefs, and current nutrition practices on the nutrition intentions of participants.

## 2. Theoretical Background

Bandura’s Social Cognitive Theory, SCT [22], has been applied as a theoretical and conceptual framework for highlighting the critical role of personal factors such as knowledge, skills, and self-efficacy beliefs in predicting nutrition-related intentions and behavior of adults [23,24]. Knowledge refers to an individual’s comprehension level, whether factual or applied [25], regarding the information, principles, and action strategies necessary to execute a behavior [26]. Understanding the risks and benefits of nutrition is a fundamental prerequisite for nutritional behavioral intentions and decision-making. People can only intend to change their behavior when they understand its potential benefits for them [22,25]. As such, the development of nutrition-related intentions is fundamentally rooted in nutrition knowledge [27,28].

Knowledge is “a modifiable determinant of behavior” [29]. Gardner and colleagues [30] found in their sample of pregnant women that knowledge of nutritional benefits correlated with participants’ intentions to increase fruit and vegetable consumption and reduce high-fat and high-sugar intake. Also, nutrition information available using a mobile app mitigated barriers to self-perceived personal limitations for healthy eating and promoted healthy eating behaviors [31]. Likewise, an increased understanding of nutrition and more favorable attitudes towards the benefits of eating fruits and vegetables promoted intentions to take steps toward improving dietary habits [32]. A systematic review of 23 Social Cognitive Theory-based studies of fruit and vegetable intake revealed that knowledge is one of the most consistent variables predicting nutrition behavior [33].

While nutrition knowledge is a foundational basis for building nutrition intentions and facilitating behavior change, it is essential that this knowledge is integrated with motivational and action-oriented strategies to maximize effectiveness [25]. To connect nutrition knowledge to nutrition intentions and behavior, nutrition education programs often include skill-building elements such as cooking classes and meal planning workshops. A qualitative study exploring nutrition- and food-related interventions in SUR settings reported that nutrition knowledge gained through nutrition education and hands-on cooking skill-building had a positive influence on participants’ food-related attitudes and behaviors [34]. Another study of the impacts of a nutrition education-based cooking series found that the intervention significantly improved participants’ knowledge, confidence and desired dietary habits [35]. Similarly, in a SCT-based quantitative study that provided participants with education on food resource management and healthy cooking, Patel and colleagues reported a positive association between the intervention and participants’ improved confidence and satisfaction in their meal planning skills [36]. These participants used their new nutrition skills to empower themselves to improve their nutritional practices [35,36]. By enhancing culinary skills, participants are better equipped to make healthier dietary choices in their daily lives [37].

Bandura’s SCT also emphasizes the role of self-confidence and self-efficacy in facilitating nutrition intentions. Self-efficacy beliefs refer to a person’s ability or self-confidence to plan and implement the actions necessary to address future situations. These beliefs affect whether individuals believe they can achieve success, the level of effort they exert, and their perseverance when encountering challenges [22,25,26,27]. Self-efficacy is a robust and consistent predictor of behavioral intentions across various health behavior domains, including nutrition [7,24,38]. Schwarzer et al. reported positive associations between self-efficacy and nutrition intentions and noted that individuals with high levels of self-efficacy are more likely to convert their intentions into actions [5]. In a study examining SCT predictors of dietary behavior and physical activity among patients with type-two diabetes, self-regulation was a strong predictor of dietary behavior, and both self-regulation and self-efficacy were strong predictors of dietary behavior and physical activity [8]. Self-efficacy has also been noted as an essential mediator of the links between intentions and actual behavior change. If self-efficacy is not present, intentions alone are not likely to lead to behavior change [5].

In addition to the SCT personal variables, research has also identified past and current behaviors as strong predictors of intentions and behavior [39]. The development of intentions is shaped by past experiences and ongoing behavioral trends, especially when those actions are prominent and evoke strong emotions [40]. A previous study reported that existing dietary habits (e.g., baseline fruit and vegetable consumption) were a predictor of the intention to adopt healthier eating in a sample of first- and second-year psychology undergraduate students [41].

## 3. Materials and Methods

### 3.1. Program Description

Data for this explorative study come from a preliminary evaluation of Healthy Choices for Your Recovering Body (HCYRB), a nutrition education curriculum developed by the University of Kentucky Nutrition Education Program for limited-resource adults in substance use recovery. The HCYRB curriculum aims to improve participants’ nutrition intentions; understanding of the role of nutrition and physical activity in recovery; physical activity level; consumption of water, regular soda, and energy drinks; confidence to make nutritious choices; cooking confidence; and food resource management confidence. The HCYRB curriculum and its core components have been described elsewhere [13]. Briefly, HCYRB consists of seven lessons, all of which integrate nutrition and physical activity with recovery concepts, nutrition content, and food preparation skills. The lesson topics include “Moving Toward Good Health”, which discusses balanced eating and physical activity and common physical issues for those in recovery (i.e., gut health issues); “Cooking for Better Health”, which emphasizes the relationship between food preparation skills, eating nourishing foods, and SUR; and “Keeping Food Safe”, which teaches the food safety concepts of clean, separate, cook and chill. Other lessons include “Building Healthier Meals with MyPlate”, which provides MyPlate guidance for meal planning and preparing one-dish meals using grains; “Using Food Labels to Guide Your Choices”, focused on reading Nutrition Facts labels and ingredient lists to choose foods; and “Shifting Toward Healthier Choices”, which emphasizes the importance of limiting saturated fat, added sugars, sodium, and caffeine while providing tips for preparing and purchasing healthy snacks. The final unit, “Eating Better on a Budget”, teaches participants skills for planning, shopping, and preparing meals on the USDA’s Thrifty Food Plan. Each lesson features an engaging participant handout, facilitator’s guide, and interactive activities including physical activity, food preparation, and mindfulness practices.

To enhance curriculum delivery, HCYRB educators received training on best practices for working with SUR audiences, including the use of trauma-informed approaches and relational trust development; instructional design implementation and curriculum delivery; and foundational content on nutrition and physical activity, food safety practices, and food preparation skills. Educators also receive an implementation guide that provides background information on substance use and recovery demographics, commonly used terms in recovery settings, avoiding stigmatizing language, and a summary of literature on the importance of nutrition and physical activity in substance use recovery.

### 3.2. Data Collection and Description of Participants

Evaluation plan for HCYRB employed a single-sample survey design where HCYRB participants completed the voluntary program evaluation survey in person at the end of their participation in the program. The paper-based survey assessed the extent to which HCRYB improves participants’ understanding of the role of nutrition and physical activity in their recovery; current physical activity level; consumption of water, regular soda, and energy drinks; confidence to make nutritious choices; cooking confidence; and food resource management confidence. The HCYRB curriculum development and program evaluation team developed the survey. However, items measuring current nutrition and physical activity practices were borrowed from a previous study [42]. A team of dieticians and nutrition experts from the University of Kentucky reviewed survey questions and scales for content validity to ensure alignment with the HCYRB curriculum.

HCYRB program participants are typically 18 years or older, male or female, of limited resources, and enrolled in an HCYRB nutrition class at a substance use recovery center. All HCYRB participants were invited to complete the program evaluation survey and there were no exclusion criteria. The data collection protocol for the program evaluation/quality improvement study was reviewed by the Institutional Review Board at the University of Kentucky and was deemed non-human subject research and did not require written consent. A total of 2163 HCYRB participants completed the post-participation evaluation survey. As shown in Table 1, the sample was predominantly Caucasian (86%), male (54%), 30–39 years old (32%) and with some college education (36%).

### 3.3. Variables and Measurements

As detailed below, we conducted a reliability analysis using Cronbach’s alpha (α) to test the internal consistency of all summated rating scales. We also conducted exploratory factor analysis via principal component analysis to ensure that all items load appropriately onto the constructs they measure, thereby enhancing the measurement validity of the constructs.

We measured participants’ nutrition intentions as a summed scale (α = 0.93) consisting of 8 items asking participants to indicate their intentions to practice nutrition behavior when they have a choice (e.g., “I intend to use MyPlate to make choices for meals,” “I intend to choose healthy snacks,” “I intend to eat more servings of fruit every day,” “I intend to eat more servings of vegetables every day,” and “I intend to choose foods that are high in fiber”). Response categories for the items ranged from “strongly disagree” = 1 to “strongly agree” = 4. Principal component analysis indicates that all the items load into a single factor with factor loadings ranging from 0.75 to 0.86. Of note, the evaluation tool asked about participants’ intentions “when they have a choice” because most of the participants were in residential treatment facilities where they could not choose their own diet and nutrition (except for water and snacks, and drinks from vending machines when available on site).

In line with the tenets of SCT [22], the survey solicited information about participants’ demographic characteristics, knowledge and skills, self-efficacy beliefs, and current behavior. Demographic variables included gender (male vs. female), race/ethnicity (recoded as white vs. others, given the predominance of Caucasians in the sample), age, and educational attainment. Table 1 presents the demographic characteristics of participants.

Bandura’s Social Cognitive Theory [22] emphasizes the role of knowledge and skills in the development of behavioral intentions. Research demonstrates that people are more likely to intend to change their behavior when they know of the risks and benefits and perceive that they have the skills to make a change [22,23,24,25,26,32]. Hence, measures of nutrition knowledge and skills in this study included participants’ understanding of the importance of physical activity, which was assessed with a single item, “I understand why physical activity is important in my recovery,” with response categories ranging from “strongly disagree” = 1 to “strongly agree” = 4. Likewise, participants’ understanding of the importance of nutrition in their recovery was measured with the single item “I understand why nutrition is important in my recovery,” with response categories ranging from “strongly disagree” = 1 to “strongly agree” = 4. The survey also assessed cooking skills, which were measured as a summated rating scale consisting of five items (α = 0.92) regarding participants’ self-reported skills in “using knife skills in the kitchen,” “using basic cooking skills (for example mixing ingredients or using an oven and/or stovetop),” “following recipe directions,” “measuring ingredients,” and “cooking raw meat and chicken safely.” Factor loadings for the items ranged from 0.81 to 0.90.

According to SCT, self-confidence and self-efficacy are positively associated with intentions. As such, the HCYRB evaluation survey also included measures of self-efficacy, specifically, self-reported food resource management confidence and self-perceived confidence in choosing nutritious foods. Based on the existing literature [43,44,45], we conceptualized food resource management confidence as participants’ self-reported belief and confidence in their ability to stretch their food dollars and make healthy food choices within limited budgets. As such, we measured food resource management confidence using a five-item scale (α = 0.92) that asked participants to indicate their level of agreement/disagreement with statements regarding their confidence to “budget enough money for food purchases,” “check for sales on foods before I shop,” “plan my meals before I shop for groceries,” etc., with factor loadings ranging from 0.82 to 0.89. In the same vein, we measured self-perceived confidence to choose nutritious food with a four-item scale (α = 0.91) that asked participants to indicate their agreement/disagreement with statements regarding their confidence “that I have the knowledge to choose/prepare healthy snacks or meals,” “that I have the ability to choose/prepare healthy snacks or meals,” etc., with factor loadings ranging from 0.86 to 0.89. Response categories for all scale items ranged from “strongly disagree” = 1 to “strongly agree” = 4.

Finally, SCT identifies current behaviors as factors predicting future intentions. In line with SCT, the HCYRB survey solicited information about participants’ current nutrition and physical activity behaviors. Participants’ current physical activity level was measured by the self-reported number of days per week that participants exercise for at least 30 min. Participants’ current frequency of water consumption of water was assessed with the question, “How often do you drink at least 6 cups of water every day?” (response categories ranged from 1 = “never” to 6 = “always”). Likewise, participants’ current frequency of regular soda consumption and frequency of energy drinks consumption were each measured with one item, “How often do you drink regular sodas, not diet?” and “How often do you drink energy drinks?” Response categories for the items ranged from 1 = “never” to 7 = “four or more times a day.”

### 3.4. Statistical Analysis

We estimated linear regression models to examine the predictive effects of personal factors (knowledge and skills, self-efficacy beliefs and current nutrition and physical activity practices) on participants’ nutrition intentions. We included these covariates in the models based on the tenets of the SCT model, our guiding framework for this study, which clearly stipulates the critical role of these personal factors in nutrition-related intentions [5,25,26,37].

Specifically, we tested two nested linear regression models. Model 1 included food resource management confidence, cooking skills, confidence to choose nutritious foods, current nutrition practices (i.e., water, soda, and energy drink consumption) and physical activity behaviors, and understanding of the importance of nutrition and physical activity in recovery as predictors while Model 2 included all the predictors in Model 1 and controlled for the effects of demographic variables (i.e., age, gender, education, and race). We compared Models 1 and 2 by assessing and comparing R^2^ change (and its significance) between the two models using the R^2^ Change F-Test.

All analyses were conducted in SPSS (version 31, IBM Corp., Armonk, NY, USA; 2025). Statistical significance was set at *p* < 0.05 for all estimated independent variables and missing cases were removed from the analysis.

## 4. Results

In terms of model fit, the adjusted R^2^ for Model 1 was 0.360, indicating that Model 1 explained about 36% of the variability in participants’ intentions. As shown in Table 2, the results of Model 1 showed statistically significant positive effects of participants’ cooking skills (B = 0.09, *p* = 0.017) on participants’ intentions. Likewise, the effects of the two measures of self-efficacy, i.e., food resource management confidence (B = 0.57; *p* < 0.001) and confidence to choose nutritious foods (B = 0.20; *p* < 0.001), were positive and statistically significant. With regard to the effects of current behaviors, the results revealed statistically significant positive effects of frequency of water consumption (B = 0.48; *p* < 0.001), frequency of soda consumption (B = 0.25; *p* < 0.001), frequency of energy drink consumption (B = 0.21; *p* = 0.007), and current physical activity level (B = 0.14; *p* < 0.001). However, the results showed that participants’ understanding of the importance of nutrition to recovery and their understanding of the importance of physical activity to recovery were not statistically significant predictors of nutrition-related intentions.

The adjusted R^2^ for Model 2 was 0.387, indicating that Model 2 explained approximately 39% of the variability in participants’ nutrition intentions (Table 2). Model 2 included all the variables in Model 1 and controlled for participants’ demographic variables, specifically, age, gender, and education. Results of an R^2^ change F-test comparing Models 1 and 2 showed a small but statistically significant R^2^ change of 0.03 (*p* < 0.001), indicating that the inclusion of the demographic variables enhanced the robustness of Model 2. Hence, we selected Model 2 as the final model for interpretation, given that it has slightly better fit indices and includes control variables to help tease out potential confounding effects.

As shown in Table 2, after introducing demographic variables into the Model, the results of Model 2 revealed that the effects of the two measures of self-efficacy, i.e., food resource management confidence (B = 0.58, *p* < 0.001) and confidence to choose nutritious foods (B = 0.31, *p* < 0.001), remained positive and statistically significant. Likewise, the results showed that the effects of current behaviors, i.e., current frequency of water consumption (B = 0.47, *p* < 0.001), frequency of soda consumption (B = 0.25, *p* < 0.001), frequency energy drink consumption (B = 0.19, *p* = 0.018) and current physical activity level (B = 0.15, *p* < 0.001) remained positive and statistically significant. However, after the introduction of the demographic variables, the effect of cooking skills, which elicited statistical significance in Model 1, became non-significant in Model 2.

Of the demographic variables introduced in Model 2, only gender elicited a statistically significant effect (B = 1.31, *p* < 0.001), which was in favor of male participants. As shown in Table 2, age, educational attainment and race did not emerge as statistically significant predictors of nutrition intentions among participants in HCYRB. Finally, as observed in Model 1, the knowledge variables (i.e., participants’ understanding of the importance of nutrition to recovery and their understanding of the importance of physical activity to recovery) did not achieve statistical significance in Model 2.

## 5. Discussion

This study examined the relationships between personal factors and the nutrition intentions of participants in HCYRB, a nutrition education program for limited-resource adults in substance use recovery. Overall, the results align with previous studies that reported positive associations between measures of self-efficacy and nutrition intentions [5,23,24]. Specifically, we found that having a high level of confidence in one’s ability to manage their food resources and confidence to choose nutritious foods are positively associated with nutrition-related intentions among HCYRB participants. These results are not surprising given that self-efficacy supports the development of nutrition intentions [5,24]. That is, individuals are more likely to intend to make a nutrition change (and sustain nutrition behavior) when they feel like they have the confidence to do so. There may be more to learn from this result in future studies by exploring the reasons why participants felt confident in their ability to manage their food resources and choose nutritious foods. Potential findings may inform future efforts and programmatic adjustments to support improving confidence and self-efficacy in other HCYRB content areas.

Our findings also align with existing research linking current behavior with future practices [38,39,40]. We found that current levels of water consumption and physical activity level are positively associated with future nutritional intentions, indicating that HCYRB participants wish to continue these practices beyond their recovery journey. Moreover, we found positive effects of current levels of soda consumption and energy drink consumption on nutrition intentions. Given that HCYRB teaches tips for reducing consumption of soda and energy drinks and emphasizes the importance of shifting to beverages that limit added sugars and caffeine [13], we speculate that these findings mean that participants who currently consume higher amounts of these drinks also have a strong desire for change. Of note, HCYRB emphasizes the importance of healthy drinks based on research linking consumption of regular soda and energy drinks to increased risk for initial substance use, relapse and/or dependency [46,47,48,49]. However, these findings may also indicate that HCYRB was not effective for curbing the intentions of heavy consumers of soda and energy drinks. Given that our study is exploratory and cross-sectional, these explanations are speculative, and further research and more rigorous evaluations of HCYRB are needed to further clarify the relationship between nutrition intentions and current consumption of soda and energy drinks. Findings from such an effort could inform practical changes to program implementation and/or instructional design.

In our study, gender was a significant predictor with male HCYRB participants showing statistically significantly higher nutrition intentions than female participants. While we cannot speculate on the reasons for this finding, previous research using self-reported variables indicates that males have a greater tendency to overreport or provide more socially desirable responses on surveys [50,51,52]. To ensure participants are supplying the most accurate responses and increase the validity of self-reported data, future HCYRB programming may emphasize the implementation of cognitive and situational approaches that support the validity of self-reported data before administering program evaluation surveys to participants [52]. In practice, this includes clarifying confusing questions, reassuring participants personal data will be kept confidential, and explaining that there are no consequences for how questions are answered.

Our study is not without limitations. First, while SCT typically explores the direct and indirect roles of personal and social factors, our study only examined the direct effects of personal factors. Per SCT principles, the effects of personal factors on participants’ nutrition intentions do not occur in isolation from social factors such as family support [24]. Indeed, the moderate R^2^ value of 0.39 indicates that there are other factors or variables associated with HCYRB participants’ nutrition intentions that were not included in the model. Like much of the extant literature on the topic, our model did not assess the efficacy of specific components of the HCYRB intervention on impacting participants’ nutrition intentions or behaviors. Future studies exploring factors associated with the nutrition intentions of adults in SUR may use more advanced statistical methods, such as path analysis and structural equation modeling, to explore the direct and indirect effects of personal and social factors and the effects of specific elements of targeted interventions. Additionally, future studies should consider a longitudinal design to better evaluate the transition of participants’ nutrition intentions into sustained behavior changes over time.

Our sample for the analyses consisted of participants in a single program, HCYRB. The experiences of these participants may not be reflective of all limited-resource adults in SUR. As such, the findings may not be generalized to other studies. Relatedly, HCYRB participants were not clustered in the same location but at different sites across the state. Our regression analyses did not account for the differences in geographical locations and potential heterogeneity due to receiving the curriculum at different sites. Future evaluations of HCYRB may employ advanced statistical analysis, such as multilevel linear models, to account for potential variances due to geographical locations.

## 6. Conclusions

This study examined the personal factors associated with the nutrition-related intentions of participants in a nutrition education program for limited-resource adults in SUR. Specifically, the study conducted linear regression models to examine the effects of nutrition knowledge and skills, self-efficacy beliefs, and current nutrition practices on the nutrition intentions of participants. The results showed statistically significant effects of measures of self-efficacy, specifically, food resource management confidence and confidence in choosing nutritious foods; participants’ current level of water consumption, soda consumption, energy drink consumption and physical activity level; and gender. The effects of participants’ cooking skills and knowledge variables (i.e., participants’ understanding of the importance of nutrition to recovery and their understanding of the importance of physical activity to recovery) were not statistically significant.

In line with the tenets of SCT, the findings of this study underscore that self-confidence and current positive health behaviors, specifically water consumption and physical activity, are significant predictors of nutrition intentions. The findings of this study can inform programs aiming to improve nutrition intentions and behaviors of limited-resource adults in SUR. Future programs may focus on building participants’ nutrition confidence while in treatment, as these variables predict intentions to adopt nutrition-behavior change after treatment and at reentry into society. Specifically, future nutrition education programs should consider implementing instructional methods and content that enhance self-efficacy and increase motivation. To do this, future SUR nutrition education programs should consider implementing consistent strategies throughout the program, so participants stay motivated, monitor personal progress, and develop confidence. Embedded strategies that strengthen efforts to support nutrition intentions include goal setting activities for all outcome objectives with specific measurable steps; providing ample instructional activities that promote skill development practice with repeat exposure that builds toward greater complexity; addressing barriers to outcomes using practical and relevant strategies that reflect the situational needs of the participant; and creating learning environments that foster participant confidence through educator feedback and peer social support. Implementing these strategies addresses programmatic structures and support systems that support intentionality.

While SUR nutrition education programs provide foundational knowledge to increase participants’ awareness and recognition of how the information impacts or is related to recovery, knowledge alone is insufficient to drive behavior change; nutrition knowledge must be paired with activities that build motivation to reinforce self-confidence, which in turn promotes nutrition intentions and eventual behavior change.

Moreover, future SUR nutrition education programs may implement strategies to promote and affirm participants’ current positive nutrition behaviors that support recovery. In our study, participants with high levels of physical activity and water consumption reported higher nutrition intentions. As participants master these healthy behaviors throughout the nutrition education process and/or during their recovery, they observe meaningful physical health benefits that further strengthen their confidence and promote motivation toward continuing the behavior.

## Figures and Tables

**Table 1:** Demographic characteristics of HCYRB participants (*n* = 2163).

Demographic Characteristics	Frequency	Percentage
Sex		
Female	932	43.1
Male	1176	54.4
Missing/Not provided	55	2.5
Age Group		
18–29	449	20.8
30–39	687	31.8
40–49	559	25.9
50–59	244	11.3
60 and above	59	2.6
Missing/Not provided	165	7.6
Race		
White	1865	86.2
Other	239	11.0
Missing/Not provided	59	2.7
Education		
Did not complete high school	454	20.9
Completed high school/GED	519	24.0
Some college	776	35.9
Graduated from a 2-year college	84	3.9
Graduated from a 4-year college	63	2.9
Postgraduate	15	0.7
Missing/Not provided	252	11.7

**Table 2:** Regression models for the effects of personal factors and nutrition intentions (*N* = 2163).

Independent Variables	Model 1			Model 2		
	B Coefficient	95% CI	*p* Value	B Coefficient	95% CI	*p* Value
Constant	8.30 ***	6.58–10.02	<0.001	4.32 ***	2.38–6.26	<0.001
Measures of Knowledge						
Understanding of the importance of nutrition to recovery	−0.13	−0.53–0.78	0.680	0.20	−0.46–0.86	0.546
Understanding of the importance of physical activity to recovery	0.39	−0.80–0.49	0.201	−0.19	−0.82–0.44	0.556
Cooking Skills	0.09 *	0.01–0.17	0.017	0.08	−0.004–0.15	0.063
Measures of Self-Efficacy						
Food resource management confidence	0.57 ***	0.50–0.68	<0.001	0.58 ***	0.49–0.70	<0.001
Confidence to choose nutritious foods	0.20 ***	0.22–0.51	0.001	0.31 ***	0.17–0.46	<0.001
Measures of Current Behavior						
Frequency of water consumption	0.48 ***	0.33–0.57	<0.001	0.47 ***	0.35–0.58	<0.001
Frequency of soda consumption	0.25 ***	0.11–0.33	<0.001	0.25 ***	0.15–0.36	<0.001
Frequency of energy drink consumption	0.21 **	0.05–0.37	0.007	0.19 *	0.03–0.35	0.018
Current physical activity level	0.14 ***	0.05–0.20	<0.001	0.15 ***	0.08–0.23	<0.001
Age	-	-	-	−0.09	−0.23–0.05	0.189
Gender (Male vs. Female)	-	-	-	1.31 **	1.02–1.61	<0.001
Educational attainment	-	-	-	<0.01	−0.89–0.89	0.999
Race (Caucasian vs. Others)	-	-	-	−0.44	−0.90–0.03	0.066

Note: *** = statistically significant at *p* < 0.001; ** = statistically significant at *p* < 0.01; and * = statistically significant at *p* < 0.05. Model 1: R^2^ = 0.364; adjusted R^2^ = 0.360. Model 2: R^2^ = 0.392; adjusted R^2^ = 0.387.

## Data Availability

Data for this study is not publicly available due to privacy or ethical restrictions. However, readers interested in seeing the data can submit a formal data request to the University of Kentucky’s Nutrition Education Program.

## Abbreviations

The following abbreviations are used in this manuscript:

HCYRB	Healthy Choices for Your Recovering Body
SUR	Substance Use Recovery
